# Influence of broodmare aging on its offspring’s racing performance

**DOI:** 10.1371/journal.pone.0271535

**Published:** 2022-07-21

**Authors:** Sota Inoue

**Affiliations:** 1 Graduate School of Environmental Studies, Nagoya University, Nagoya, Japan; 2 Wildlife Research Center, Kyoto University, Kyoto, Japan; University of Florida, UNITED STATES

## Abstract

Maternal aging has negative influences on the development and racing performance of their offspring in racehorses. However, the mechanism by which pregnancy at old age reduces the race performance of the offspring is unknown. Here, two hypotheses were posited: 1) Foals born to older mares are more likely to have muscular, skeletal, and cognitive disadvantages (direct effects). 2) Foals born to older mares are more likely to be affected by non-physiological factors correlating with the mare’s age, such as the quality of sires (e.g. low-quality sires are likely to be chosen as partners of older broodmares). To test these hypotheses, the effect of the broodmare’s age on the offspring’s racing performance was examined, while controlling for the effects of the stallion’s quality, age, and ID, offspring’s sex, trainer, and the location of the training center. Information of racehorses registered to the Japan Racing Association were collected from the Japan Bloodhorse Breeders’ Association website. Overall, results showed that the racing performance of horses born from older mares was lower than that of horses born from younger mares. However, generalized linear mixed models (GLMM) indicated that the quality of sires was significantly associated with the offspring’s racing performance, rather than the broodmare’s age itself. Furthermore, the age of broodmares was negatively correlated with the quality of sires, although the variance inflation factor was low. Therefore, the effect of maternal aging was negligible or only limited, and rather, the sire’s quality had an important influence on the offspring’s racing performance. Low quality sires, or cheap stallions in other words, are likely to be chosen as partners of older blood-mares, which may have reproductive risks such as lower fertility and higher rate of miscarriages. The present study suggests that the conventional belief that racehorses born from older mares show lower performance may not always be accurate.

## 1. Introduction

Information about parents is an important attribute of racehorses. The racing ability and future value of a racehorse in the breeding business is estimated from information such as its parent’s racing performance, the rarity of bloodlines, and the compatibility between maternal and paternal lines. Many studies have reported the effect of heritability on the racing performance using various indicators, such as speed and stamina ratings, best race distance, time, handicap, and earnings [1, 2]. For example, the heritability of earning is estimated around 0.2–0.4, which is a moderate value, yet sufficient to facilitate selection [2].

Among the factors that determine the price of a racehorse is its parents’ age [3]. In general, maternal aging has been shown to have a negative effect on offspring health, lifespan, and stress resistance in various taxa [4–6]. These negative aspects of maternal aging have been also reported in Thoroughbred horses, i.e. racehorses. In several studies, maternal age was reported to be related to lower fertility, higher embryonic loss, mortality and morbidity in neonatal foals [7–10].

In addition to fetal development, maternal aging is considered to have negative consequences on the offspring’s racing performance. A racehorse’s timeform rating, an example of speed ratings, peaks when its mother is 9 years old, after which it declines [11]. Similarly, negative relationships between maternal age and the likelihood of winning stakes races (the highest class of races) have been reported [12, 13].

During particular life phases, such as pregnancy and lactation, female animals experience heightened physiological demands and adapt to consequent changes. In equine species, as well as other mammalian species, mares and newborn foals are physiologically unstable for the entire perinatal period. The dynamics of hormones leading to the delivery and lactation drastically change energy metabolisms in foaling mares. In newborn foals, crucial adaptive processes to the extrauterine environment occur; these include the transition from fetal to adult circulation, the onset of pulmonary respiration, and enteral nutrition. Many studies on this topic have been published [14–18], however there is scant data on the influence of broodmare aging on its offspring’s racing performance presented in literature.

The detailed relationship between maternal aging and an offspring’s racing performance is still unclear. There are two possible ways in which maternal age can affect offspring’s racing performance: 1) through direct effects and 2) through indirect effects. First, foals born to older mares may be more likely to have muscular, skeletal, and cognitive disadvantages (i.e. direct physiological effects). Second, breeding farms avoid mating older mares with high performance sires because of beliefs that older females have higher risks during reproduction (e.g. lower fertility, higher chances of miscarriages, etc.) [3]. Instead, low-quality sires are likely to be chosen as partners to older mares. In other words, a broodmare’s age may be indirectly reducing the offspring’s racing performance through lowered sire quality. These two hypotheses are not exclusive to each other. In fact, it is possible that factors in the former hypothesis may cause the situation explained in the latter.

In the present study, the two hypotheses were statistically tested to determine which of these could better explain the phenomenon. If the first hypothesis holds true, maternal age would influence the racing performance of their offspring when the quality of the sire is controlled. If the second hypothesis holds true, there would be no effect of the broodmare’s age on their offspring’s racing performance. In addition, it is necessary to find evidence that low quality sires are likely to be chosen as partners of older broodmares.

## 2. Materials and methods

### 2.1 Data collection

Information on 17,885 Thoroughbred horses, born between 2001–2010 and registered to the Japan Racing Association (JRA), were collected online. The following pieces of information about each racehorse were gathered: sex, trainer, location of the training center, the birth year of the sire and broodmare, breeding farm, region of the breeding farm, earnings, total number of races, number of races won, and the quality of the sire. The sire’s quality was quantified by the average earnings index (AEI), a metric calculated by the mean earnings of its offspring. The mean of each AEI between 1995–2015 was used for each sire. When the AEI was not available in the dataset, their offspring were excluded from analyses. Castrated horses and horses, which had not experienced any races, were also removed from the dataset.

Information on 15,461 individuals (10,902 males and 4,559 females) remained and were used for subsequent analyses. The data included 9,686 broodmares, whose ages ranged from 2–25 years old (mean = 9.43, sd = 3.89) and 695 sires, 3–27 years old (mean = 10.22, sd = 3.92).

### 2.2 Data analysis

All data analyses were conducted in the R (3.5.1.) environment. First, the relationships between the age of broodmares (ABM), and the age of sires (AS) and sires’ AEI were investigated using Spearman’s correlation tests [19]. Second, the relationship between ABM and the racing performance of offspring was tested.

The normality of ABM, AS, and the number of winning races (NW) which was used as an indicator of racing performance was tested using Kolmogorov-Smirnov tests. None of the three variables followed a normal distribution (ABM: D = 0.998, p < 0.0001; AS: D = 1.0, p < 0.0001; NW: D = 0.5, p < 0.0001). NW was used as an indicator of racing performance because it requires lower computational load compared to earnings. In fact, the number of races won was highly correlated with earnings in the entire dataset (S = 9.31e^10^, rho = 0.849, p < 0.0001). Because the effect of ABM on offspring performance could appear gradually or only after a certain age [15], ABM was analyzed both as a continuous and categorical variable. When ABM was treated as a categorical variable, horses 16 years old and younger were treated as young (N*young* = 14608) and 16 years old and older as old (N*old* = 853). This threshold was determined by a previous study, which showed that pregnancy rates decrease and embryonic death rate increase in broodmares 14–18 years old [20].

The following is a general outline of the analysis. Generalized linear mixed models (GLMMs) with hurdle models were used to test the hypotheses that ABM affects the racing performance of their offspring. Ten sub-datasets containing 3,000 individuals’ information from the entire dataset were made to run the GLMMs because the computational load was too high when the entire dataset was used. To construct the sub-datasets, 300 horses per year (2001–2010) were randomly sampled to construct one dataset. Some horses were in multiple datasets.

**Fig 1:**
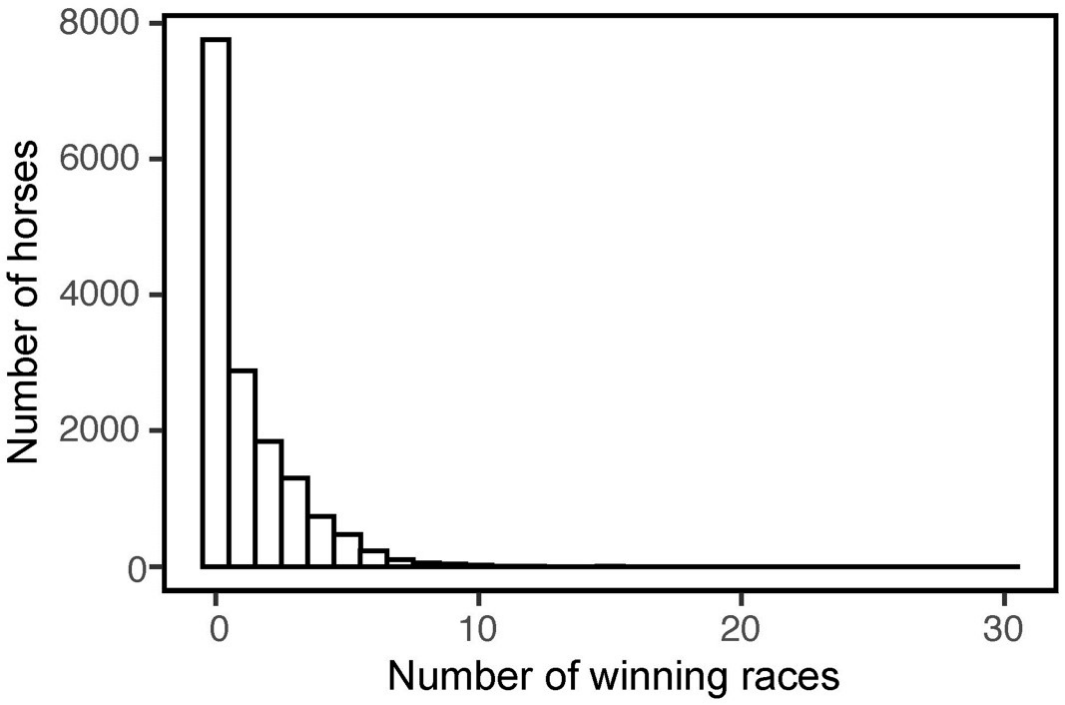
Relationship between the number of horses and the number of winning races (N = 15,461).

A hurdle model was used to explain NW as the response variable because it seemed to be zero-inflated [21] (Fig 1). Hurdle models, also known as two-parts models, evaluate zero and non-zero counts independently for modeling the zero-inflated counts [22]. The first part is a binary model, where the response variable corresponds to whether the response variable is positive or zero. If the response variable is not zero, it moves on to the second part. The second part is the zero-truncated model, which is usually a zero-truncated Poisson or negative binomial model. In the present study, a zero-truncated Poisson distribution was used. This two-parts structure seemed to match the characteristics of the dataset in this study. Each race in the JRA has its own classification of horses that can participate, with grades and age limits. Owing to these limitations, the number of horses whose NW > 0 are constrained by the number of races with the grade. Further, the time given until a horse wins its first race and the time given until it wins its second race and onwards is largely different. This is because the JRA requires horses to win at least one race by the beginning of September at three years old (it varies from year to year), or else they will essentially lose their rights to participate in future races. As long as a horse wins one race by this time, it is able to participate in races even if it does not win for several years. Therefore, the time given to win the first race is much shorter than the time given to win the second race and onwards. Given these features of the dataset, it was suitable to conduct separate regressions for samples with NW of zero and more than zero.

Two GLMMs for the regression in the hurdle model were constructed. In the first model, the effect of ABM was assumed to increases linearly. Thus, ABM was introduced as a continuous variable into the model. For the purposes of this paper, this model will be referred to as the “continuous model”. In the second model, the effect of ABM was introduced as categorical scale, namely “old” and “young”, as mentioned earlier. This model was named the “transition model”. In both models, the other explanatory variables were AS, AEI, and sex. ABM in the continuous model and AS were divided by 10 to match the AEI scale. The variance inflation factors (VIF) among the fixed effects were smaller than 1.02 when calculated using “vif” function in R package, “car” [23]. The information on trainers, ID of the sires, and the production farm were included as random effects. Logit and log link function were used for the Bernoulli and zero-truncated Poisson distributions, respectively.

Each coefficient in the GLMMs were estimated using the Markov chain Monte Carlo method with No U-turn sampler (NUTS). The “brms” package [24], which allows fitting Bayesian linear models using “Stan”, in R version 3.5.1., was used. Four chains of 10,000 iterations and 7,000 warm-ups were set up. Weakly informative normal distributions (mean = 0, sd = 100), were used as prior distributions of each parameter. After the sampling, all parameters’ $\hat{R}$ were ≦ 1.01. 1,000 samples were obtained from each dataset and 10,000 samples were used in total. Posterior distributions of each sub-dataset showed similar results, but biases were detected for each dataset (Supporting information (SI) 3). Therefore, it was better to mix the results of each sub-dataset, instead of relying on one sub-dataset. The significance of each parameter was determined by whether the 95% credible interval of the mixed posterior distribution was across 0.

## 3. Results

### 3.1 Correlations between ABM, and AS and AEI

Correlations between ABM, and AS and AEI were tested. No correlation was found between ABM and AS in the full-dataset (S = 6.09e^9^, rho = 0.011, p = 0.19). On the other hand, a significant correlation was found between ABM and AEI in the full-dataset, although the correlation coefficient was low (S = 6.28e^11^, rho = -0.02, p = 0.015). There was a significant difference in AEI between the two age categories of ABM (Brunner-Munzel Test Statistic = -5.6635, df = 949.76, p < 0.001, Brunner-Munzel Test) (SI 1 in S1 File). These indicate that stallions with lower AEI were selected as breeding partners for older mares.

### 3.2 Winning races and parents’ age

**Fig 2:**
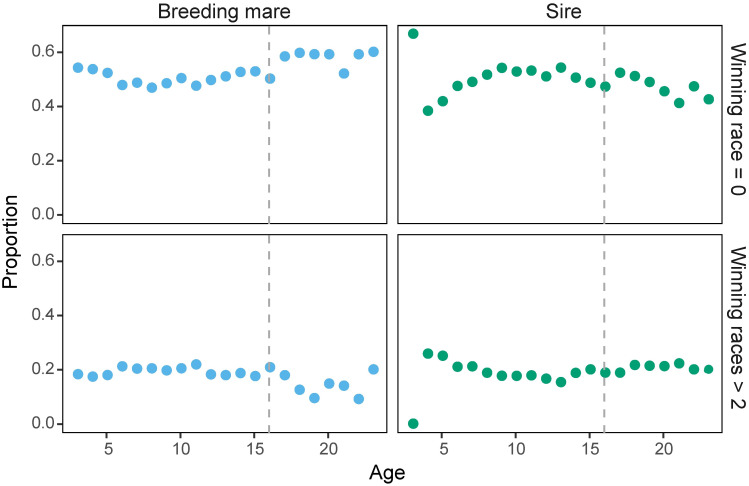
Relationship between the racing performance of offspring and parents’ age. The upper row shows the proportion of offspring which did not win any races. In the lower row the proportion of offspring which won more than two races are drawn. The dashed line indicates 16 years old, which was determined as a threshold of age category. This figure contains results for parents over the age of 3 and under the age of 24. A figure including all data is shown in SI 2 in S1 File.

As ABM increased, the proportion of offspring that did not win any races (NW = 0) increased (S = 665.72, p = 0.0072, rho = 0.568, the Spearman’s correlation test) (Fig 2, SI 2 in S1 File), and the proportion of offspring which won more than two races decreased (S = 2278, p = 0.029, rho = -0.479, the Spearman’s correlation test). On the other hand, no clear relationships between NW (offspring with NW = 0 or NW > 2) and AS were found (NW = 0, S = 1980, p = 0.209, rho = -0.286; NW > 2, S = 1245.6, p = 0.407, rho = 0.191) (Fig 2).

### 3.3 The effects of the age of broodmares and sires

**Fig 3:**
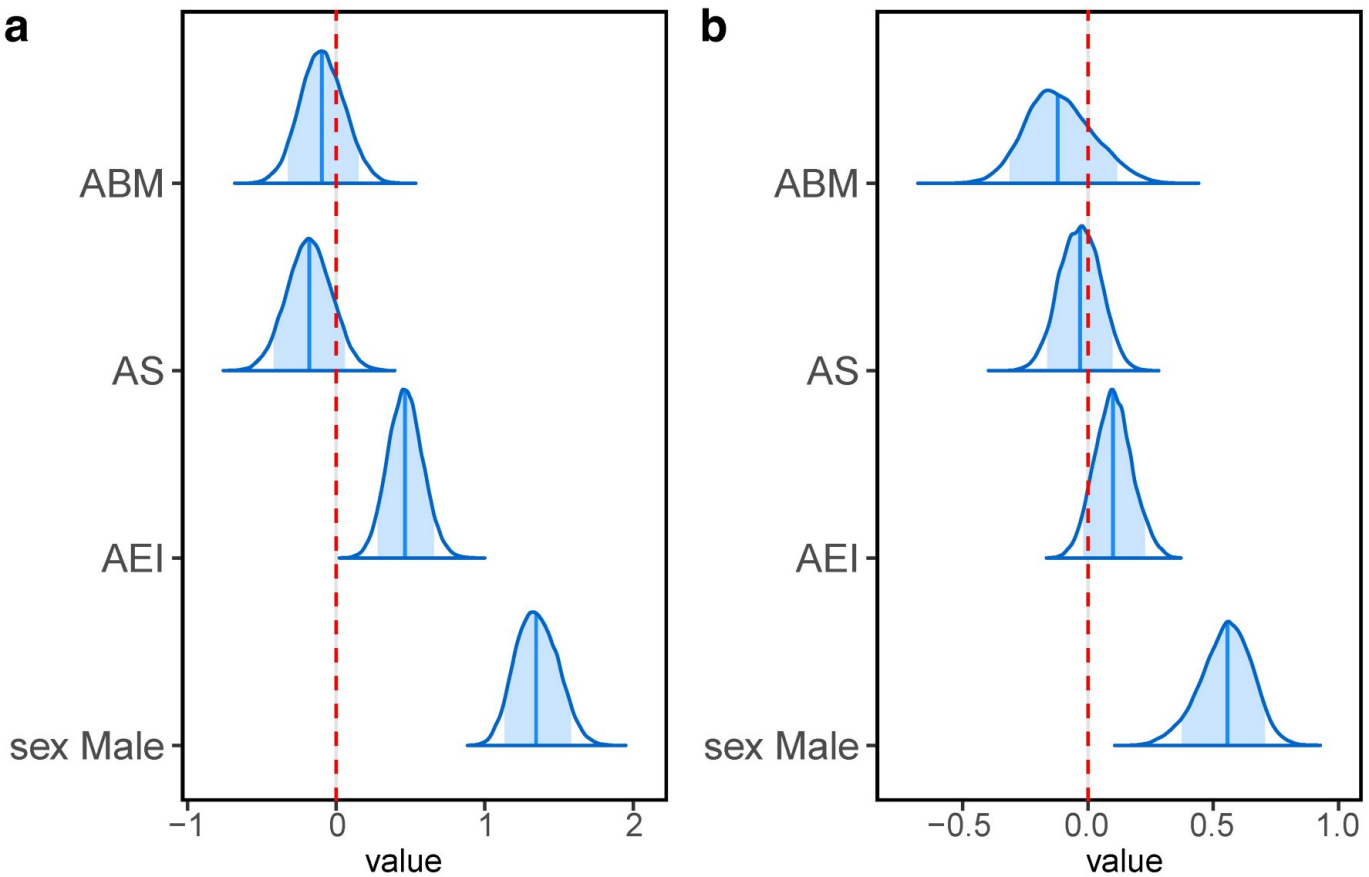
Estimated posterior densities of each parameter in the continuous model. 95% of posterior distributions are drawn here. Edges of distributions indicate the lower bound of 2.5% and upper bound of 97.5%. The dark blue vertical lines indicate the median. The dark blue area in each distribution shows the high density interval (HDI) which includes 89% of samples. The red dashed line indicates zero.

To examine whether ABM affects the racing performance of their offspring, GLMMs with hurdle models were used. In the continuous model, results of GLMMs showed no significant effect of ABM on their offspring’s racing performance in either parts (1st part, median: -0.095, 95% CI: -0.377–0.204; 2nd, median: -0.121, 95% CI: -0.354–0.166) (Fig 3, Table 1). AS was not significant either (1st part, median: -0.180, 95% CI: -0.474–0.115; 2nd, median: -0.031, 95% CI: -0.191–0.125) (Fig 3, Table 1). On the other hand, AEI showed a significant positive effect on the offspring’s racing performance in the 1st step, but not in the 2nd step (1st part, median: 0.465, 95% CI: 0.240–0.703; 2nd, median: 0.100, 95% CI: -0.043–0.255) (Fig 3, Table 1).

**Table 1:** Results of GLMM.

Parameter^(1)^	Lower CI (2.5%)	Lower HDI (5.5%)	Median (50%)	Upper HDI (94.5%)	Upper CI (97.5%)
**Model1—first**					
ABM	-0.377	-0.324	-0.095	0.15	0.204
AS	-0.474	-0.419	-0.18	0.06	0.115
AEI	0.24	0.28	0.465	0.658	0.703
Male	1.091	1.135	1.346	1.581	1.632
**Model1—second**					
ABM	-0.354	-0.312	-0.121	0.116	0.116
AS	-0.191	-0.162	-0.031	-0.097	0.125
AEI	-0.043	-0.018	0.1	0.227	0.255
Male	0.329	0.374	0.557	0.706	0.739
**Model2—first**					
Young	-0.120	-0.039	0.335	0.709	0.790
AS	-0.455	-0.4	-0.169	0.072	0.124
AEI	0.229	0.269	0.452	0.641	0.686
Male	1.077	1.119	1.327	1.56	1.608
**Model2—second**					
Young	-0.357	-0.289	0.089	0.39	0.45
AS	-0.188	-0.158	-0.023	0.11	0.138
AEI	-0.038	-0.013	0.099	0.224	0.251
Male	0.323	0.367	0.552	0.702	0.735

^(1)^ ABM: age of breeding mare, AS: age of sire, AEI: average earning index

**Fig 4:**
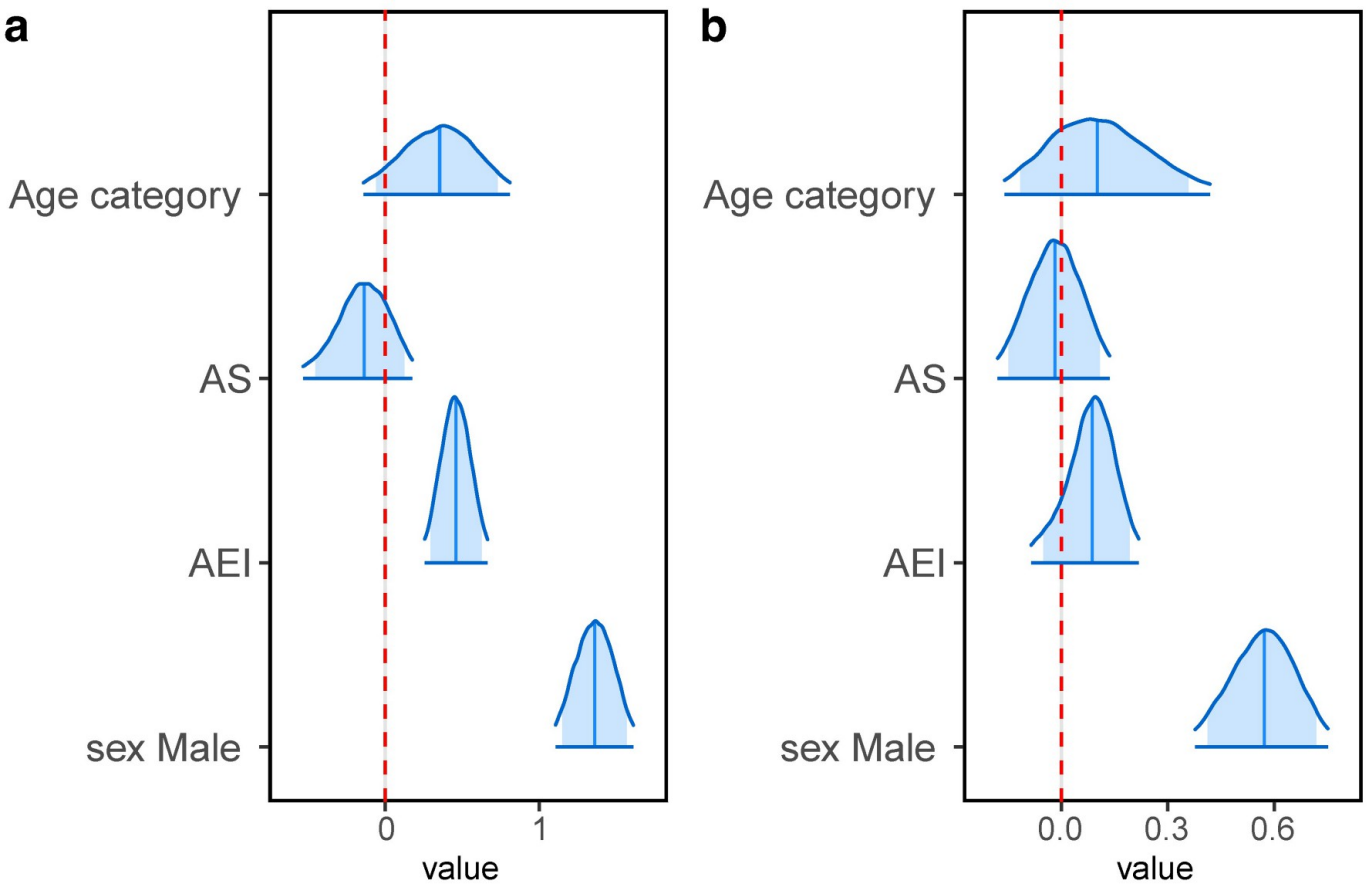
Posterior densities of each parameter in the transition model. 95% of posterior distributions are drawn here. The edges of distributions indicate the lower bound of 2.5% and upper bound of 97.5%. The dark blue vertical lines indicate the median. The dark blue area in each distribution shows the high density interval (HDI) which includes 89% of samples. The red dashed line indicates zero.

In the transition model, no significant effect of the age category of broodmare were found in either steps, as was the case for the continuous model (1st part, median: 0.335, 95% CI: -0.120–0.790; 2nd, median: 0.089, 95% CI: -0.357–0.450) (Fig 4, Table 1). AS was not significant either (1st part, median: -0.169, 95% CI: -0.455–0.124; 2nd, median: -0.023, 95% CI: -0.188–0.138) (Fig 4, Table 1). AEI was found to have a positive effect in the 1st step, but not in the 2nd step (1st part, median: 0.452, 95% CI: 0.229–0.686; 2nd, median: 0.099, 95% CI: -0.038–0.251) (Fig 4, Table 1).

## 4. Discussion

Based on several previous studies, it has been generally considered that a broodmare’s age (ABM) is negatively correlated with their offspring’s racing performance [11–13, 25]. However, the mechanisms underlying this phenomenon were unclear. The following two hypotheses were tested in this study. (1) Direct effects: foals born to older mares are more likely to have muscular, skeletal, and cognitive disadvantages, and thus perform poorly in races. (2) Indirect effects: older mares are more likely to be chosen as mates of low-quality sires, resulting in lower offspring performance.

In the present study, a negative correlation between offspring’s racing performance and ABM was found in racehorses in Japan, when other factors were excluded from analyses. However, GLMMs found that ABM had no significant effect on the racing performance, whereas AEI, i.e., quality of sires, had a significant positive effect. In addition, AEI was negatively correlated with ABM. These three results suggest that the negative correlation between maternal aging and offspring’s racing performance is likely to be spurious because of the confounding factor, AEI. This supports the second hypothesis: it is likely that maternal aging does not biologically affect the racing performance of their offspring, but rather it is the effect of sires with lower AEI with which the aged broodmares mate with, that result in lower racing performance of the offspring.

The present study shows that the effect of maternal aging is negligible or limited, if the offspring is healthy enough to make a debut as a racehorse. Indeed, the study does not necessarily prove the absence of negative effects caused by maternal aging on the development of the offspring. For example, it is possible that horses with mental and/or physical disabilities have already been eliminated during their training stage or the symptoms only emerge at older age, so the effect is not apparent in this dataset. If a horse with a relatively mild disability is not allowed to debut, the effect of maternal aging will be close to zero; on the other hand, if a horse with a severe disability is allowed to run, the effect may be reflected strongly. Therefore, there still is a possibility that the biological and direct effects of maternal aging impact an offspring’s racing performance (hypothesis 1). It is also possible that these biological effects are real, and are causing breeding farms to choose low quality sires as older broodmare’s mates (hypothesis 2). Potential differences in the breeding-training systems and decision-making of trainers and owners are better to be noted in future comparative studies among countries to understand the relationship between maternal age and the racing performance of their offspring.

The present study revealed that the age of the broodmares did not directly influence the racing performance of offspring, thus it is not a reliable piece of information for racing predictions. It is suggested that the quality of sires, which decreases as broodmare’s age increases, lowers the performance of offspring. Further studies are needed to fully understand the influences of maternal aging. Breeding records including information on mating, fertility rates, embryonic death rates, and development should be analyzed, although these factors are not available in the web pages used in the present study. Unverified relationships, speculations, and rumors are common in sports [26]. Some of them are scientifically valid while others are invalid or more complex than expected. It would be interesting to examine these speculations using statistical methods—the results may reveal facts that cannot be predicted by one’s intuition.

## 5. Conclusion

In this study, the detailed relationship between maternal aging and the racing performance of offspring in racehorses in Japan was revealed. The statistical analysis with 10 years of data suggested that the negative correlation between maternal aging and offspring’s racing performance seems to be spurious because of the confounding factor, AEI. Thus, it is likely that maternal aging does not biologically affect the racing performance of their offspring, but rather it is the effect of sires with lower AEI with which the aged broodmares mate with, that result in lower racing performance of the offspring. This study provided a new perspective on the effect of maternal aging on race performance of foals and the complex system of horse breeding. In the future, it would be possible to conduct similar studies with data sets from other countries. This extension will unveil further details of the mechanisms and potential diversity of breeding systems of racehorses.

## Supporting information

S1 File (PDF)
