# Supplementary material for: Influence of broodmare aging on its offspring’s racing performance
Source: PLoS One. 2022 Jul 21;17(7):e0271535. doi: 10.1371/journal.pone.0271535 (PMC9302849; doi:10.1371/journal.pone.0271535)

Supporting information

Influence of broodmare aging on its offspring's racing performance

Sota Inoue[1,2]

1: Graduate School of Environmental Studies, Nagoya University, Japan

2: Wildlife Research Center, Kyoto university, Japan

SI 1: Boxplot of AEI in each age category.

AEI of young and old female.

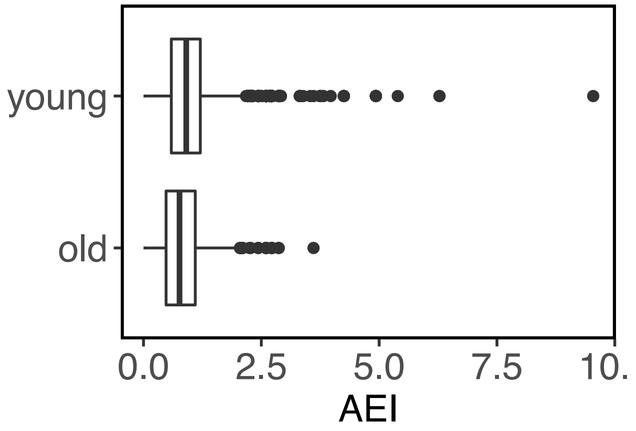

SI 2: Relationship between the racing performance of offspring and parents' age.

In upper row, the proportion of offspring which did not win any races and in lower row, those of offspring which won more than two races are drawn. Dashed line indicates 16 years old which was determined as a threshold of age category.

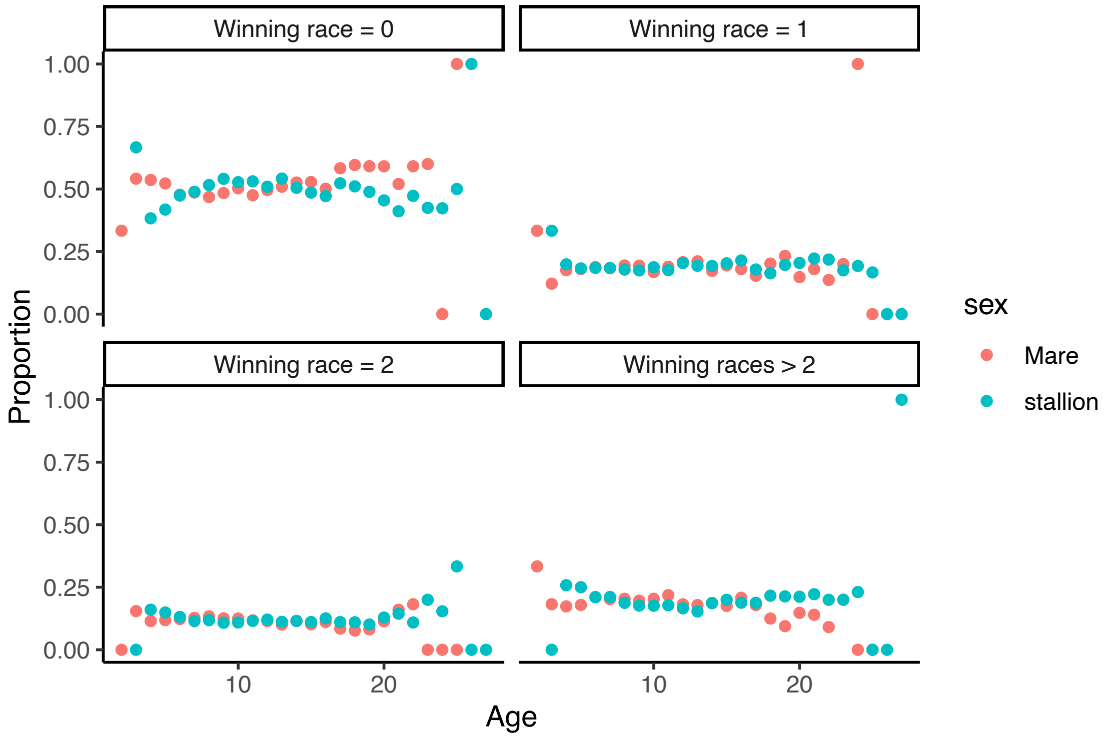

SI 3a: Posterior density of each parameter in each session in continuous model.

Posterior density of GLMM in continuous model. There are 10 distributions.

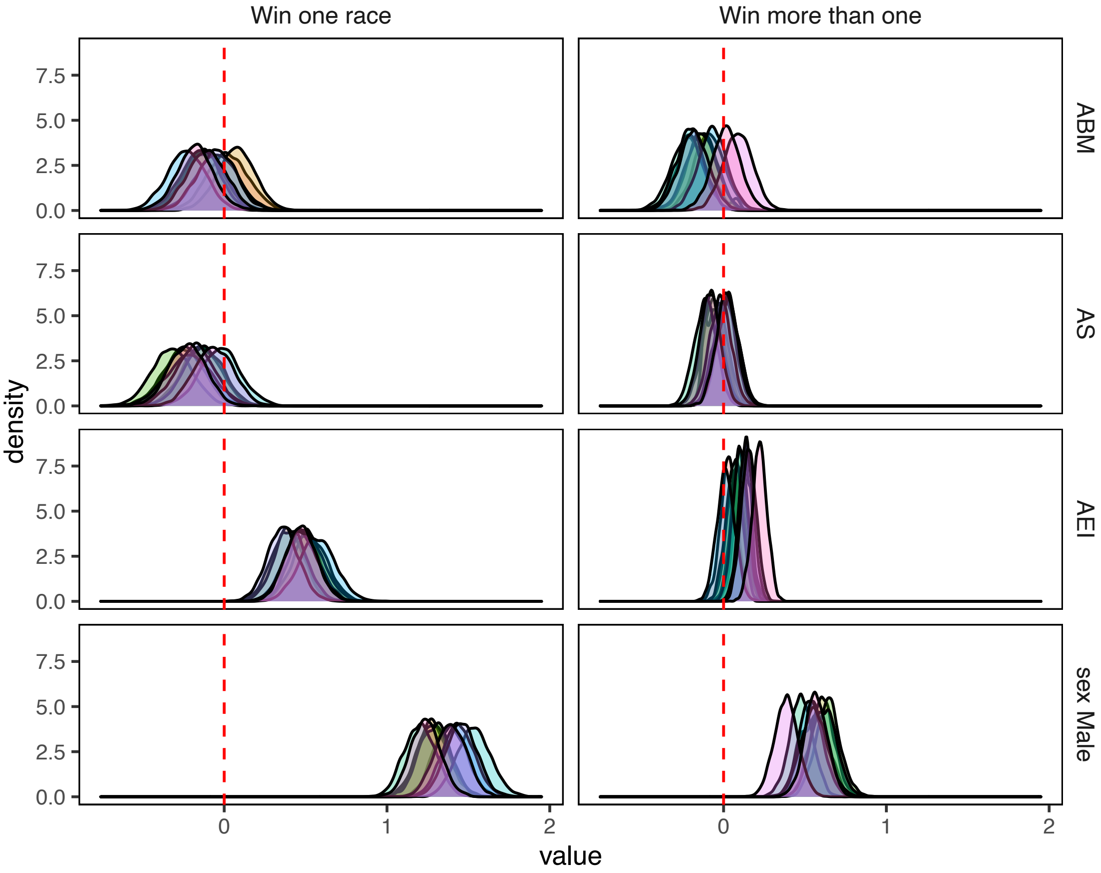

SI 3b: Posterior density of each parameter in each session in the transition model.

Posterior density of GLMM in transition model. There are 10 distributions.

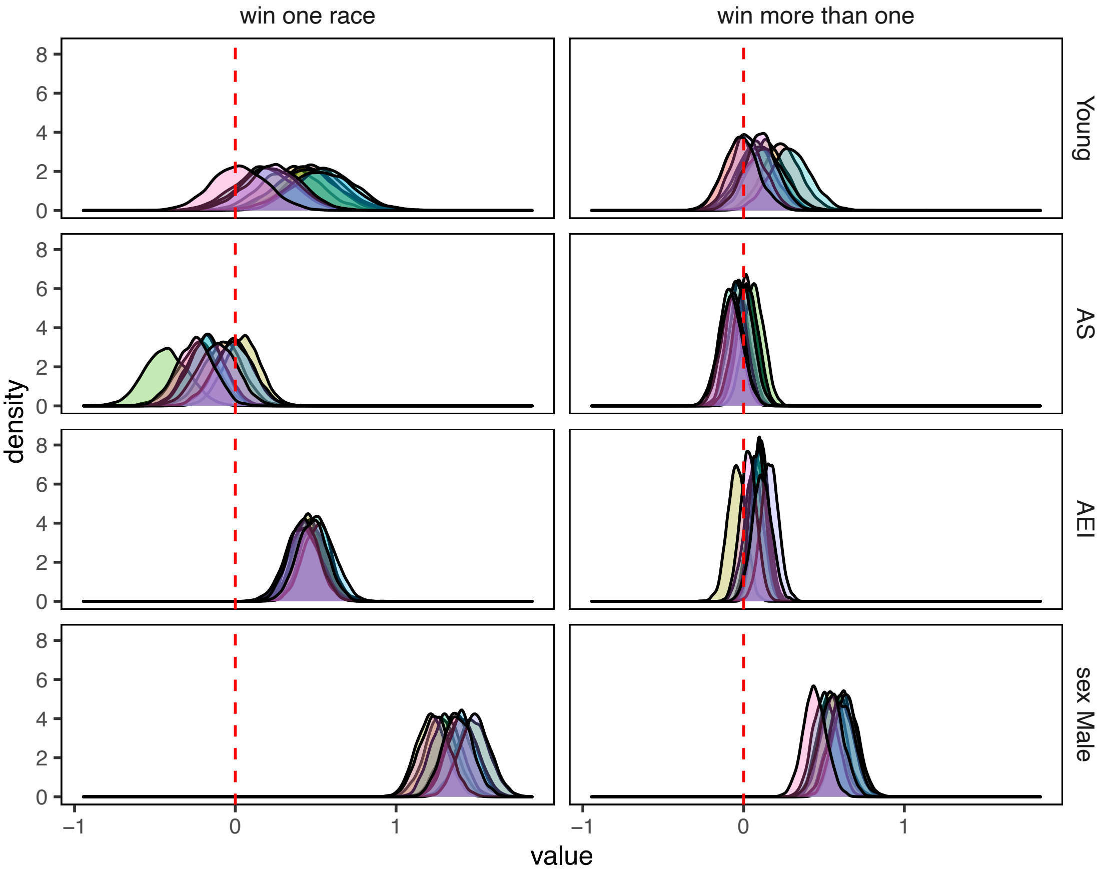

Supplement: S1 File — (PDF) [file pone.0271535.s001.pdf]
